# Supplementary material for: Conversion of Charge Carrier Polarity in MoTe2 Field Effect Transistor via Laser Doping
Source: Nanomaterials (Basel). 2023 May 22;13(10):1700. doi: 10.3390/nano13101700 (PMC10222443; doi:10.3390/nano13101700)
Supplement: Supplementary file 1 [file nanomaterials-13-01700-s001.zip › nanomaterials-2377428-supplementary.pdf]

Communication

Conversion of Charge Carrier Polarity in $MoTe_2$ Field Effect Transistor via Laser Doping

Hanul Kim [1], Inayat Uddin [2], Kenji Watanabe [3], Takashi Taniguchi [4], Dongmok Whang [1,5,*] and Gil-Ho Kim [1,2,*]

[1] Sungkyunkwan Advanced Institute of Nanotechnology (SAINT), Sungkyunkwan University (SKKU), Suwon 16419, Republic of Korea; hanulk@skku.edu
[2] Department of Electrical and Computer Engineering, Sungkyunkwan University (SKKU), Suwon 16419, Republic of Korea; inayatuddin@skku.edu
[3] Research Center for Functional Materials, National Institute for Materials Science, 1-1 Namiki, Tsukuba 305-0044, Japan; watanabe.kenji.aml@nims.go.jp
[4] International Center for Material Nanoarchitectonics, National Institute for Materials Science, 1-1 Namiki, Tsukuba 305-0044, Japan; taniguchi.takashi@nims.go.jp
[5] Department of Advanced Materials Science and Engineering, Sungkyunkwan University (SKKU), Suwon 16419, Republic of Korea
* Correspondence: dwhang@skku.edu (D.W.); ghkim@skku.edu (G.-H.K.)

Citation: Kim, H.; Uddin, I.; Watanabe, K.; Taniguchi, T.; Whang, D.; Kim, G.-H. Conversion of Charge Carrier Polarity in $MoTe_2$ Field Effect Transistor via Laser Doping. *Nanomaterials* **2023**, *13*, 1700. https://doi.org/10.3390/nano13101700

Academic Editor: Jun Cheng Cao

Received: 17 April 2023
Revised: 4 May 2023
Accepted: 19 May 2023
Published: 22 May 2023

Copyright: © 2023 by the authors. Licensee MDPI, Basel, Switzerland. This article is an open access article distributed under the terms and conditions of the Creative Commons Attribution (CC BY) license (https://creativecommons.org/licenses/by/4.0/).

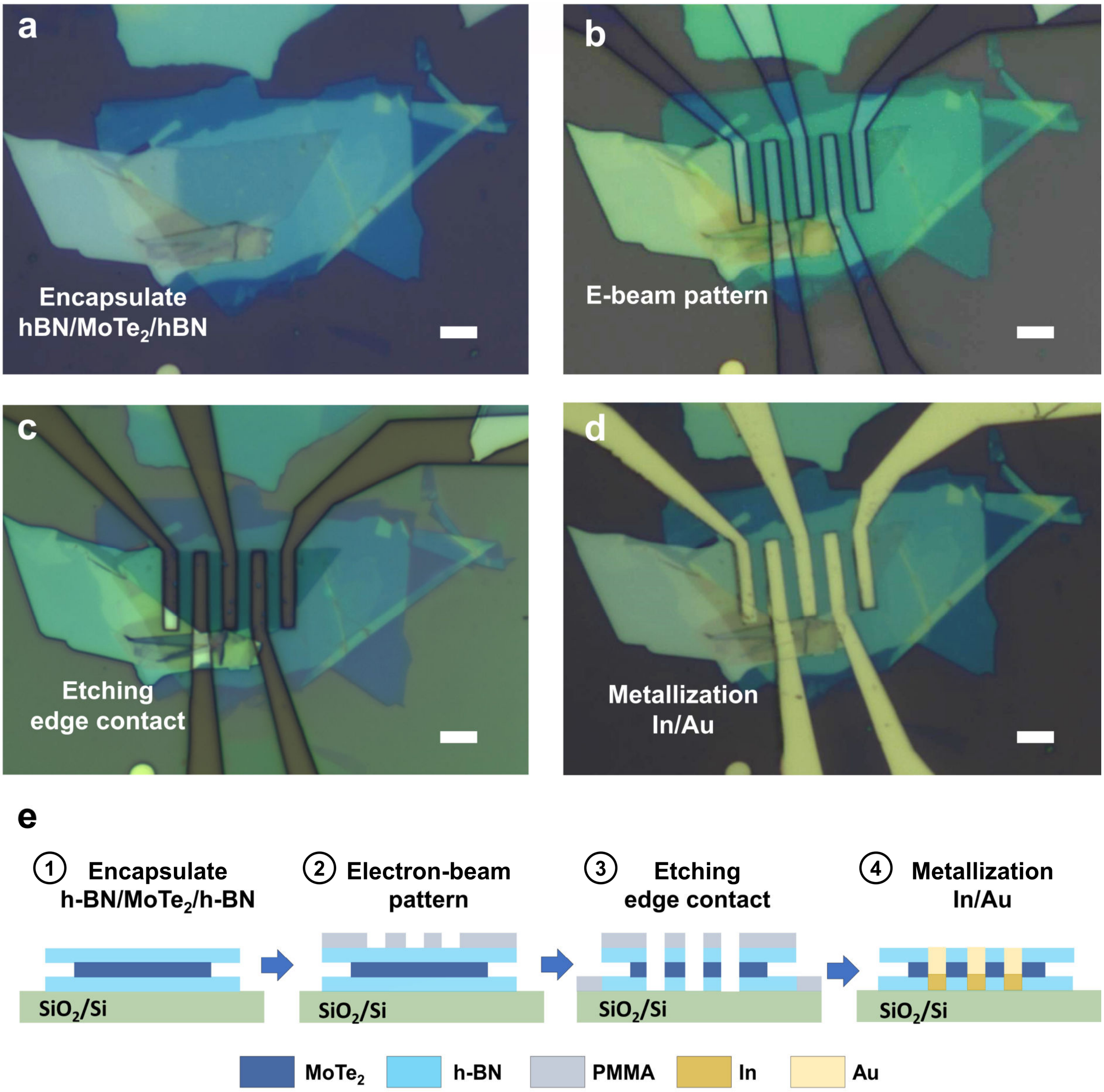

Figure S1. (a) Optical microscopy image of the first step process with encapsulated hexagonal boron nitride (hBN)/$MoTe_2$/hBN. (b) Optical microscopy image of the second step process for inner patterning with electron beam lithography. (c) Optical microscopy image of the third step process for reactive ion etching the inner. (d) Optical microscopy image of the final step process for metallization with In/Au. (scale bar 5 μm). (e) Schematic steps process for the device fabrication.

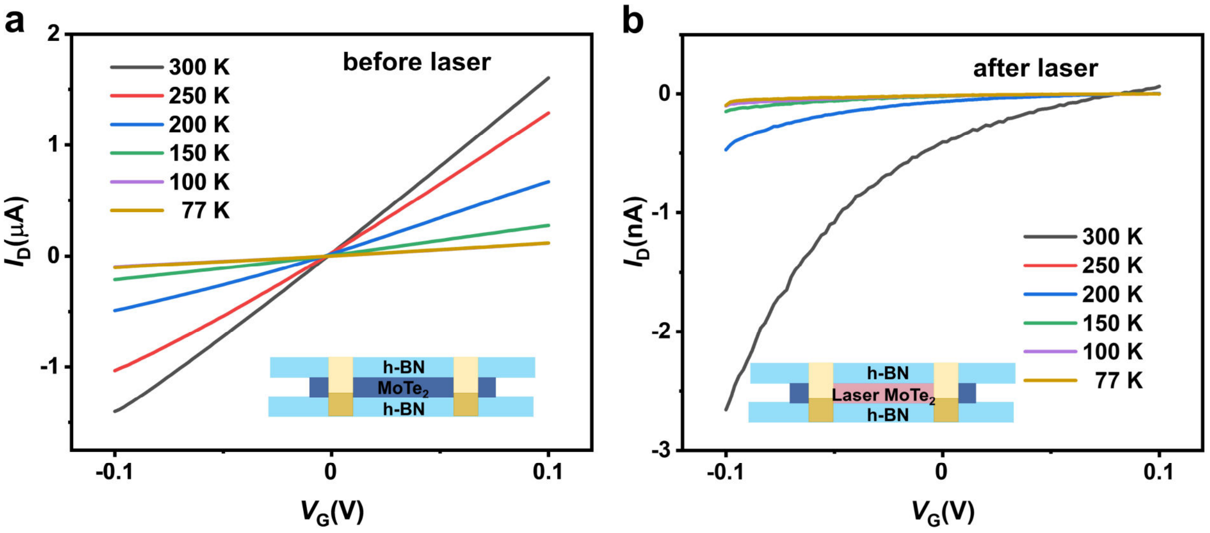

Figure S2. Comparison plot of output characteristics before and after laser at different temperatures. (a) I_D-V_D characteristics of $MoTe_2$ channel before laser scanning. (b) I_D-V_D characteristics of $MoTe_2$ channel after laser scanning.

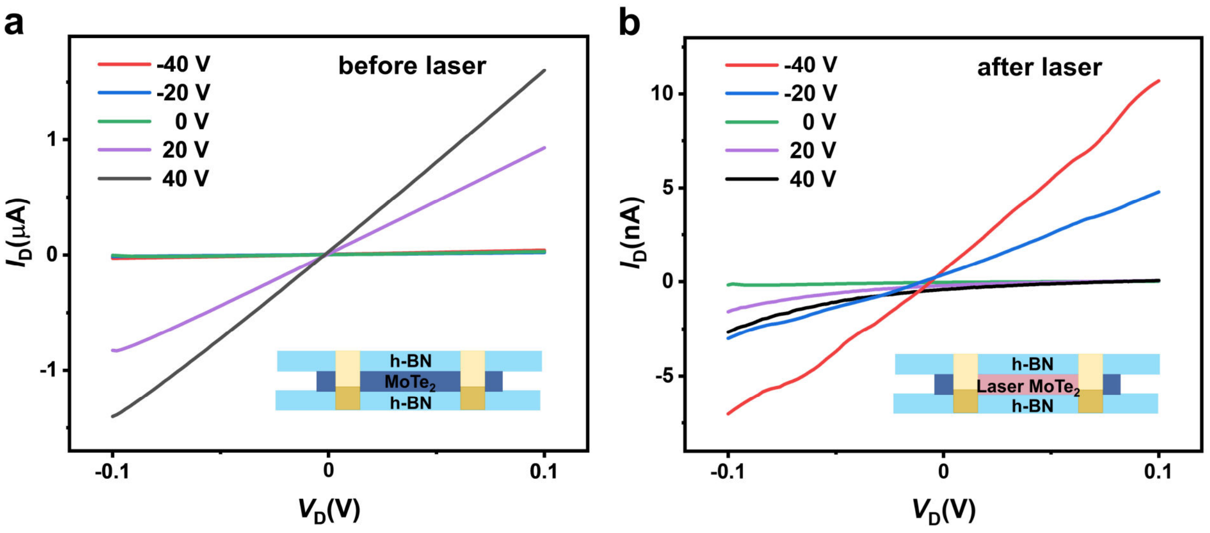

Figure S3. Comparison plot of output characteristics before and after laser at different voltage biases. (a) I_D-V_D characteristics of $MoTe_2$ channel before laser scanning. (b) I_D-V_D characteristics of $MoTe_2$ channel after laser scanning.

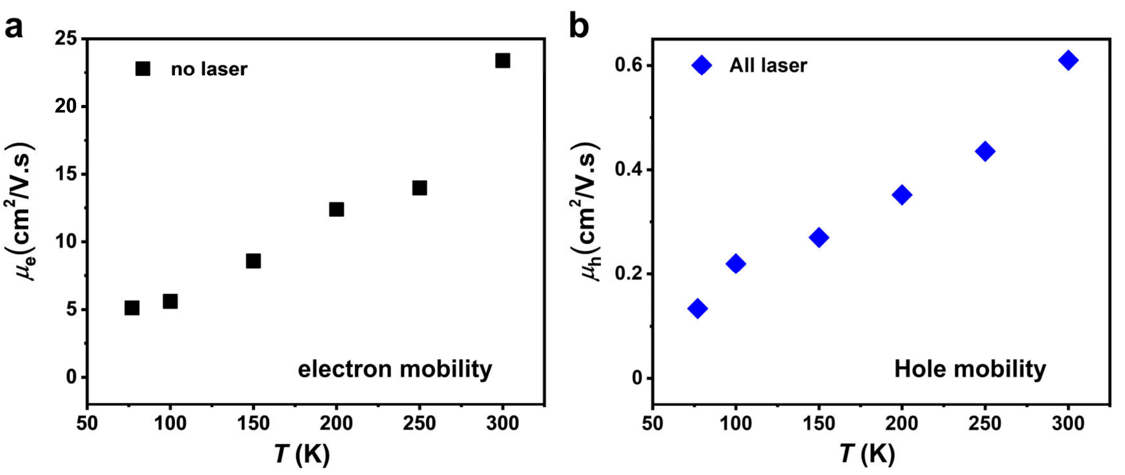

Figure S4. Comparison plot of mobility before and after laser at different temperatures. (a) Electron mobility of $MoTe_2$ channel before laser scanning. (b) Hole mobility of $MoTe_2$ channel after laser scanning.

Disclaimer/Publisher's Note: The statements, opinions and data contained in all publications are solely those of the individual author(s) and contributor(s) and not of MDPI and/or the editor(s). MDPI and/or the editor(s) disclaim responsibility for any injury to people or property resulting from any ideas, methods, instructions or products referred to in the content.
